# Annotations

**Published:** 1902-09-13

**Authors:** 


### ANNOTATIONS.

### Death of Professor Virchow.

THE death of Professor Virchow removes a figure which for almost half a century has stood before the world as a force in science, and before his country as a power in politics. Rudolf Virchow was born on October 13th, 1821. In 1843 he took his degree as *Unterarzt*, and when he was 26 years of age he became external lecturer in pathology at the University of Berlin. Then he went to Würzburg, and in the year 1856 produced his famous "Cellular Pathology," the book by which he made his mark in science. Since then he has played many parts, always with distinction; he has worked, has investigated, has taught, has been a leader in politics, a guide and reformer in municipal affairs, a man looked up to on every side. Yet to the world and to science it will always be by the illuminating work of those earlier days which led to the production of his "Cellular Pathology" that he will be remembered. Like other master minds he was able to step in at the right moment, and, partly by adding to, but more especially by logically arranging and focussing existing knowledge, he succeeded in marking an epoch. What he did was not so much to discover new facts as to lay bare the meaning of facts already known, and by insisting upon the importance of the cell as the unit of morbid, as it was already admitted to be the unit of healthy vital action, to correlate pathology and physiology. Since then, no doubt, we have advanced a good deal. The centre of interest has shifted, and the animal cell has now in turn become the stage on which still smaller cells play their part in a language only vaguely deciphered as yet by physiological chemistry. Still, great as is the light thrown by modern bacteriology upon the influences by which cell growth is modified, and firmly as the new school may hold to the faith that in the ultimate analysis all is chemistry, nothing can take away the brilliancy of that illuminating generalisation which at once made Virchow famous when he gave to the world his "Cellular Pathology."

### London Inquests.

THE proceedings of the London coroners, and the motives by which they are actuated in the holding of inquests, have been seriously called in question in the annual report of the chief officer of the Public Control Department of the London County Council. It is pointed out that there is a considerable difference in the practice of the coroners in the different parts of London in determining whether inquests should or should not be held, and, arguing from the fact that in nearly half of the inquests which were held the verdict of the jury was "death from natural causes," it is suggested that in many cases an efficient preliminary inquiry would have made them unnecessary. Much depends, of course, upon what is meant by an "efficient preliminary inquiry." It would have to be something very much more complete and authoritative than the sort of investigation now made by the coroner's officer, and one can hardly avoid suspecting that any legal machinery by which such preliminary investigations could be satisfactorily performed would be quite capable of dealing with the whole question without the intervention of the coroner and his court at all. A most serious innuendo is contained in the statement that every inducement is held out by the present law, both to the coroner and his officer, to hold inquests. We are told that if the inquest is held the coroner's officer is paid for his time, trouble and expense; but if it is not held, he not only gets no pay for his time and trouble, but actually loses any out of pocket expenses for travelling etc., incurred in making the preliminary inquiry. Similarly with the coroner: if the inquest is held it counts in his next quinquennial revision of salary, but if it is not held it does not count at all—his services go for nothing. The suggestion is obvious. Coroners are but men, and we imagine that not only ratepayers but long-suffering jurymen will agree that the holding of unnecessary inquests ought not to be encouraged in so open a fashion. As to the recommendations made by the committee for the amendment of the law, and confirmed by the Council, we can but say that they also are as open to criticism as the abuses they aim at reforming. The fact is the question is a very large one and involves the whole system of death registration—so large a one in fact that its satisfactory solution may possibly involve the abolition of the coroner and his court as at present established.

### Our Child Population.

NOTWITHSTANDING all that has been said as to the evils of over-population, no one who looks upon contemporary events from a national point of view can regard with equanimity the lowering birth-rate of this country, especially when it is contrasted with the fecundity of certain other nations with whom we find ourselves in open competition in many parts of the world. We are far better off than France where the infecundity of the race has long been a matter of national concern; our birth-rate of 28 6 per 1,000 contrasting well with theirs of 21·4. We compare badly, however, with the German Empire, which has a birth-rate of 35·6, and with Hungary with its birth-rate of 39·3; and it is unfortunately to be feared that those influences which have of late years made for a diminishing birth-rate are still extending in our midst. As a result partly of the diminishing number of births and partly of the large infantile death-rate which obtains among our town population, it appears, according to some figures given by Mr. T. A. Welton in the *Times*, that the increase in the number of children under 15 years is relatively very small—so small that we have to face a shortage of 2,092,000 children compared with the numbers which would have existed had the ratio of 1881 held good. The matter is one of enormous importance, and is perhaps more within our control than some people may imagine. Birth-rate is a matter which we cannot directly influence. But we may do much to keep alive the children who are born to us, and in view of the fact that in London alone, in one recent year, out of a total of 84,432 deaths at all ages, no fewer than 30,861 occurred among children under five years of age, it is clear that in the lessening of child mortality there is wide scope for the efforts of those who view with concern the diminishing expansiveness of our population.

